# Cutaneous leishmaniasis in Iran: A review of epidemiological aspects, with emphasis on molecular findings

**DOI:** 10.1051/parasite/2022047

**Published:** 2022-10-21

**Authors:** Zahra Nasiri, Mohsen Kalantari, Jalal Mohammadi, Salman Daliri, Davood Mehrabani, Kourosh Azizi

**Affiliations:** 1 Research Center for Health Sciences, Institute of Health, Department of Vector Biology and Control of Diseases, School of Health, Shiraz University of Medical Sciences 7153675541 Shiraz Iran; 2 Department of Health, Firoozabad Branch, Islamic Azad University 7471913113 Firoozabad Iran; 3 Clinical Research Development Unit, Imam Hospital, Shahroud University of Medical Sciences Shahroud Iran; 4 Li Ka Shing Center for Health Research and Innovation, University of Alberta, Edmonton, AB, Canada; Stem Cell Technology Research Center, Shiraz University of Medical Sciences 7134814336 Shiraz Iran

**Keywords:** Cutaneous leishmaniasis, Reservoir, Vector, Epidemiology, Systematic review, Iran

## Abstract

*Leishmania* parasites can cause zoonotic cutaneous leishmaniasis (CL) by circulating between humans, rodents, and sandflies in Iran. In this study, published data were collected from scientific sources such as Web of Science, Scopus, PubMed, Springer, ResearchGate, Wiley Online, Ovid, Ebsco, Cochrane Library, Google scholar, and SID. Keywords searched in the articles, theses, and abstracts from 1983 to 2021 were cutaneous leishmaniasis, epidemiology, reservoir, vector, climatic factors, identification, and Iran. This review revealed that CL was prevalent in the west of Iran, while the center and south of Iran were also involved in recent years. The lack of facilities in suburban regions was an aggravating factor in the human community. Some parts of southern Iran were prominent foci of CL due the presence of potential rodent hosts in these regions. *Rhombomys opimus*, *Meriones lybicus*, and *Tatera indica* were well-documented species for hosting the *Leishmania* species in Iran. Moreover, *R. opimus* has been found with a coinfection of *Leishmania major* and *L. turanica* from the northeast and center of Iran. Mashhad, Kerman, Yazd, and sometimes Shiraz and Tehran foci were distinct areas for *L. tropica*. Molecular identifications using genomic diagnosis of kDNA and ITS1 fragments of the parasite indicated that there is heterogeneity in leishmaniasis in different parts of the country. Although cutaneous leishmaniasis has been a predicament for the health system, it is relatively under control in Iran.

## Abstract (fr)

Les parasites *Leishmania* peuvent établir une leishmaniose cutanée zoonotique (LC) en circulant entre les humains, les rongeurs et les phlébotomes en Iran. Dans cette étude, les données publiées ont été collectées à partir de ressources scientifiques telles que Web of Science, Scopus, PubMed, Springer, ResearchGate, Wiley Online, Ovid, Ebsco, Cochrane Library, Google Scholar et SID. Les mots-clés recherchés dans les articles, les thèses et les résumés de 1983 à 2021 étaient leishmaniose cutanée, épidémiologie, réservoir, vecteur, facteurs climatiques, identification et Iran. Cet examen a révélé que la LC est répandue dans l’ouest de l’Iran, tandis que le centre et le sud de l’Iran sont également impliqués ces dernières années. Le manque d’équipements dans les régions suburbaines est un facteur aggravant dans la communauté humaine. Certaines parties du sud de l’Iran sont des foyers importants de LC en raison de la présence d’hôtes potentiels de rongeurs dans ces régions. *Rhombomys opimus*, *Meriones lybicus* et *Tatera indica* sont des espèces bien documentées pour héberger les espèces de *Leishmania* en Iran. De plus, *R. opimus* a été trouvé avec une co-infection de *Leishmania major* et *L. turanica* au nord-est et au centre de l’Iran. Mashhad, Kerman, Yazd et parfois des foyers de Shiraz et de Téhéran sont des zones distinctes pour *L. tropica*. Les identifications moléculaires utilisant le diagnostic génomique des fragments d’ADNk et ITS1 du parasite ont indiqué qu’il existe une hétérogénéité dans la leishmaniose dans différentes parties du pays. Bien que la leishmaniose cutanée ait été une situation difficile pour le système de santé, elle est relativement contrôlée en Iran.

## Introduction

*Phlebotomus* and *Lutzomyia* sandflies have been shown to transmit *Leishmania* promastigotes through skin bites in vertebrate hosts in the Old and New World, respectively. *Leishmania major*, *L. infantum*, *L. tropica*, *L. aethiopica*, and *L. donovani* have been demonstrated to be mainly responsible for transmission of cutaneous leishmaniasis (CL) in the Old World [97, 102, 129].

Around 12 countries in Asia, Africa, and Latin America such as Iran, Afghanistan, Pakistan, Saudi Arabia, Syria, Turkey, Algeria, Morocco, Tunisia, Colombia, Peru, and Brazil were reported as engaged states against CL [145]. From March to September 2014, 3684 cases of CL were recorded in Iran, including in Khorasan-e-Razavi (Northeast), Fars (South), and Kerman (southeast) as the most endemic regions for CL, while 31% of affected patients were below the age of 14 years [38, 72, 126].

The annual incidence of the CL from 1983 to 2013 was 30.9 per 100,000, with spread in areas with dry and desert climates in particular in the central zone of Iran [52]. These areas are plains and *Phlebotomus papatasi* is the main vector reported there [27]. *Rhombomys opimus* was illustrated as the principal host of zoonotic cutaneous leishmaniasis (ZCL) in the center and northeast of the country, *Meriones libycus* as the main vector in the south, *Meriones hurrianae* as the major host in the southeast, and *Tatera indica* as the most prevalent host in the west and south of Iran [58, 83]. Strict strategies have been undertaken to control CL in Iran, but new foci have emerged in the center, northeast, and west of Iran. Therefore, the aim of this systematic review was to define the geographical distribution of *Leishmania* spp. among human populations as well as to determine methods used for identifying *Leishmania* parasites, reservoirs, and vector hosts and the impact of environmental factors in Iran over four decades (1983–2021) to help promote interventions against CL and to decrease the disease transmission rate.

## Materials and methods

This systematic review has 5 sections including epidemiology of CL, reservoirs, vectors, climatic factors, and identification methods of *Leishmania* parasites in Iran. The authors used the preferred reporting items for systematic reviews and meta-analyses (PRISMA) standard guideline to carry out the review process and report findings.

### Search strategy and selection criteria

In this study, all related published articles were studied from 1980 up to the end of 2021. The authors searched the databases including Web of Science, Scopus, PubMed, Springer, ResearchGate, Wiley Online, Ovid, Ebsco, Cochrane Library, Google Scholar, and SID for medical subject headings (MeSH) and relevant keywords such as cutaneous leishmaniasis, epidemiology, zoonotic cutaneous leishmaniasis (ZCL), anthroponotic cutaneous leishmaniasis (ACL), *Leishmania major*, *Leishmania tropica*, Iran, rodent, *P. papatasi*, sandfly, detection, PCR, the kinetoplast, ITS, parasite, reservoir, vector, Gerbillinae, Giemsa stain, and temperature. The authors used these keywords alone or in combination through the Boolean method.

### Inclusion and exclusion criteria

All high-quality English and Persian language articles published in the world on the epidemiology of CL, reservoirs, vectors, climatic factors, and identification methods of *Leishmania* parasite in Iran were included in the study. The authors excluded articles of low quality, studies conducted in other countries of the world, review studies, meta-analyses, case reports, or series of cases.

### Quality assessment

We assessed the quality of the articles using the Strobe checklist (strengthening the reporting of observational studies in epidemiology). This checklist has 22 parts that are scored based on the importance of each section, the minimum score is 15 and the maximum is 33. In this study, the score 20 is considered acceptable [147].

### Screening and data extraction

The search results were imported into Endnote software v.x8-1 and duplicate titles were deleted. The authors entered selected studies into an abstract reading phase and checked them against the inclusion criteria. Then, the most relevant studies were selected for independent full-text reading by two researchers. The authors used a checklist to extract data from the selected studies in terms of the study location, study years, reservoir species, parasite species, DNA, and recognized method.

### Selection of articles

**Fig. 1:**
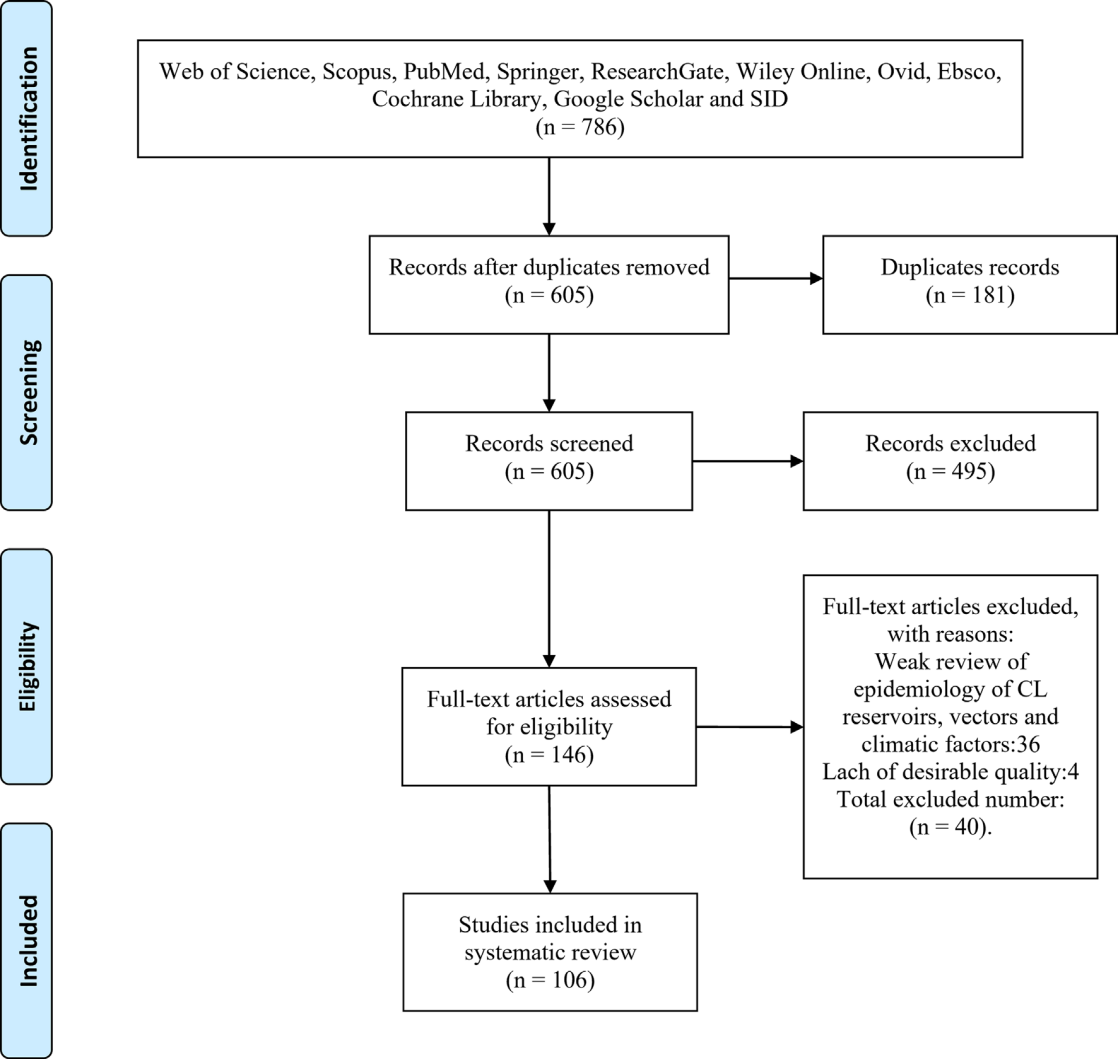
The PRISMA flow diagram.

Searching databases, the authors extracted 786 studies. First, the articles were entered into Endnote software and after an initial review, 181 articles were removed from the study due to duplication. Then, by reviewing the titles and abstracts of articles, 459 irrelevant articles were removed and after reviewing the full text of articles, 40 articles were excluded since they investigated other pathogens. Finally, 106 articles met the inclusion criteria and entered the process of systematic review (Fig. 1).

## Results and discussion

### Geographic distribution of CL

Cutaneous leishmaniasis infection has been found in all 31 provinces of Iran.

The prevalence of ZCL is high in Khorasan, Fars, and Isfahan and the highest frequency of cases has been reported from Ilam, Bushehr, and Yazd, recently. Ahvaz city in Khuzestan province and Kerman and Bam cities in Kerman province are the high endemic foci of ACL in Iran. Overall, the highest and the lowest incidence rates of leishmaniasis are reported from the western and northwest parts of the country, respectively. Two forms of ZCL and ACL occurred due to *L. major* and *L. tropica* in Iran. Fars is known as an endemic focus of CL and *Nesokia indica* or *M. libycuserythrourus* are confirmed as reservoir of ZCL [75].

Isfahan province has been a focus of CL in recent decades. Moreover, some new foci have been added to the endemic areas listed in this province. In this province, from 2011 to 2016, Isfahan, Ardestan, and Kashan cities had a high burden of CL. Kashan has been reported as the primary focus for CL in Isfahan province, while children were affected a long time ago [87, 143]. In the Aran and Bidgol districts of Kashan, 1–9-year-old children have been the target of the CL parasite [111]. In Isfahan province, the northern parts such as Borkhar and Natanz are known as hyperendemic spots and teenagers are the predominant age group affected by the diseases [59].

Yazd province is another infected region adjacent to Isfahan province in central Iran. In this province, Khatam, Yazd, and Bafgh were reported as the most problematic counties of CL [20]. Qom province has also been demonstrated as another endemic region of CL. In 2009, an outbreak was reported in this area in particular in suburban locations: Ghomrood and Ghanavat. This province is a destination for immigrants and pilgrims, but indigenous transmission is stable in this area. The majority of patients are housekeepers and cases below the age of 25 years were more commonly reported in this area [125].

In Semnan province, Damghan is an important city in central Iran concerning CL, while villages in Damghan were reported as a vulnerable area for emergence of CL disease. Most of the cases in this region did not have a previous history of traveling to endemic areas [89]. In Tehran province, CL has spread among the student population [22].

Many new CL cases are imported to different provinces of Iran from endemic areas of Pakistan and Afghanistan that are believed to be the primary source of outbreaks in some parts of the country, such as Yazd, Tehran, and Fars provinces [75]. Furthermore, in Varamin and Pakdasht, endemic districts of CL, several cases were found to be Afghani patients above the age of 20 years [22]. In Tehran province, in Pakdasht city, in Fars province, and in the south of Iran, CL is approximately distributed in all cities and towns and most frequently in Shiraz, Firouz Abad, Ghirokarzin, Farashband, Larestan, Fasa, Jahrom, Arsanjan, Kherameh, Lamerd, Zarrin Dasht, and Marvdasht [6–8, 55, 56, 74–76, 99, 113]. In this zone, all age groups were affected with CL. Also, Afghani and Pakistani immigrants comprised large parts of the cases in the south of Fars province [21].

Khuzestan province is also recognized as a hub for CL in the southwest of Iran and as a newly discovered focus. In 2014, emergence of CL was reported in Ahvaz city [70]. Moreover, this disease affected farmers and students more than any other group in the community. Susangerd, Shushtar, Behbahan, Abadan, Khorramshahr, Shush, and Shadegan cities were well documented in this province [36, 45, 63–66, 141]. In Bushehr province, southwest of Iran, the incidence of CL was 1.7 per 10,000 in Dashtestan town located in the east of Bushehr city in the winter and autumn and in September and January in 2013 and 2014 [100]. Genaveh is the other focus of CL in Bushehr province and CL is prevalent in rural areas in this city. In Dashtestan, urban areas have been at risk more than other parts [61].

In Kerman province, a comprehensive study conducted by Sharifi et al. showed that Bam, Kerman, and Jiroft cities were the high-risk areas in the southeast of Iran [136]. Orzoieh district is one of the distinct endemic areas in this province as well [77]. Hormozgan province, in the south of Iran, has been affected by malaria much more than CL; therefore, insufficient comprehensive studies have been conducted on CL in this area. Jask County in the province is confirmed as a hot spot of CL and 90% of patients were from rural localities, with an incidence of 162.5 per 100,000 population from 2006 to 2009 [37]. Another survey showed that CL was mostly spread in the southeast and northwest of Hormozgan province [4, 26]. In Sistan and Baluchestan province, southeastern Iran, a few investigations have been performed on CL. Briefly, the results revealed that CL prevalence was higher in rural (51.7%) than in urbans areas (48.3%). Cities of Zahedan (87 cases), Mirjaveh (34 cases) and Chabahar (29 cases) had the highest infection rates and Fenouj (one patient) and Nikshahr (two patients) had the lowest rates of infection. Statistical analyses showed a significant relationship between living area and the disease, and this is probably due to the presence of malaria in the province. Zahedan, Mirjaveh, and Chabahar cities in this province were infected more than any other area, and the trend of disease declined from 2009 to 2014; mostly in children below the age of 10 years [122, 132].

In Khorasan Razavi province in northeast Iran, CL is prevalent in Torghabeh–Shandiz, Sarakhs, Daregaz, and Neyshabur cities and children in these areas are affected with the disease [71, 130]. In Khorasan Razavi, Sabzevar city, children below the age of 4 years in rural areas were demonstrated to have a greater incidence of CL [151]. Like Khorasan Razavi, North Khorasan province was a hot spot of CL in the northeast of the country including Esfarayen city as a challenging region with the age group of ≥15 years most affected [110]. In this province, Jajarm town has been the main spot of CL before 2006 [9]. In Golestan province, some parts such as Kalaleh town have been confirmed as significant sites of CL. Also, in this province, the disease is prevalent in Gonbad-e-Qabus City in children below the age of five years [103]. Another city in the province is Maraveh Tappeh which is located near Iran’s northern border with Turkmenistan and is one of the hot spots of CL [28]. The north of Iran has not been confirmed as an endemic area of CL, but it can be considered an area of risk because people from neighboring provinces mostly travel to these areas [94].

In the western part of Iran, different patterns of CL have been observed. Ilam province is adjacent to Khuzestan province and is a major endemic area [91]. Ilam, Dehloran, and Mehran cities which have a common border with Iraq have a remarkably susceptible condition concerning CL because many visitors move between Iran and Iraq through the Mehran border [91, 133]. Hamedan province is another region affected by CL with an unsteady incidence rate of 1.5 per 100,000 in 2008 which increased to 12.6 per 100,000 in 2015, since the local population (about 87.1%) frequently travelled to endemic areas of CL, mainly including Ilam, Khuzestan, and Isfahan provinces. Notably, the working-age group is the main infected population. In Hamadan province, Hamadan, Bahar, and Kaboudar-Ahang cities are the main CL-infected areas [5].

In Kermanshah province, in the west of Iran, a review by Hamzavi and Khademi from 1991 to 2012 showed that the incidence rate had dramatically increased from 1.5 per 100,000 in the 1990s to 7.4 per 100,000 in 2011–2012. Also, half of the cases reported traveling to endemic areas. Qasr-e-Shirin, Eslam Abad, and Sarpol-e-zahab cities in the province are prominent foci of CL [48]. Housewives were infected in Qasr-e-Shirin city more than any other group in the community [107]. In Lorestan Province, CL has been distributed over large rural areas of Poledokhtar city in the west of Iran [10]. More investigations are needed to elucidate all aspects of CL in this province. In south Khorasan Province, Birjand and Khoosf cities located in the east of Iran were found as foci of CL with an 80% history of traveling to endemic areas [2].

### Reservoirs of CL

In Iran, several studies were carried out on host species of *Leishmania* parasites. In the central and northeastern regions of Iran, *Rhombomys opimus* (the Great gerbil) is more predominant [44, 118]. *Rhombomys opimus* has two predominant subspecies. In Golestan province, in the northwest of Iran, *R. opimus sodalist* subspecies has been reported; but in Isfahan, Semnan, Esfarayen, and Shirvan (northeast) cities, *R. opimus sargadensis* was illustrated as the prevalent subspecies [1]. In the Turkmen Sahra area, in the northeast of Iran, *R. opimus* was present with co-infection of *L. major* and *L. turanica* [86]. Also, in Golestan (northeast) and Esfahan (central) provinces, mixed infection by these parasites has been demonstrated in *R. opimus* [7].

In Isfahan city in central Iran, *L. major* zymodeme MON-26 was detected in *Meriones libycus*. It is considered an alternative host in this district [128, 149]. Additionally, in Qom province, central Iran, this species is the principal host for *L. major* in Qomrood city as an endemic area [152]. Perhaps in some parts of the northeast of Iran, such as Gonbad-e Qabus city; *M. libycus* is the main reservoir of ZCL [106]. *Meriones lybicus* is an absolute host for *Leishmania* amastigote in the south of Iran and *M. persicus* is a reservoir in some parts of this region as well. Notably, *Leishmania* amastigotes were detected in *M. persicus* for the first time in Iranian Azerbaijan in the northwest of the country [82, 85, 115–117]. In Fars province, in the south of Iran, *M. libycus, T. indica* and *Gerbillus spp.* were shown to be infected with *L.* major in Marvdash, Estahban, and Larestan cities [83, 84]. In Fars province, *L. major* has been isolated and detected by PCR assay in *Mus musculus* [104]. Also in this province, *Rattus norvegicus* has been reported as a potential host for *L. major* using isoenzyme electrophoresis and nested PCR [96]. In Jahrom, Fars Province, *R. rattus* has been shown as a reservoir for *L. major* [96].

**Fig. 2:**
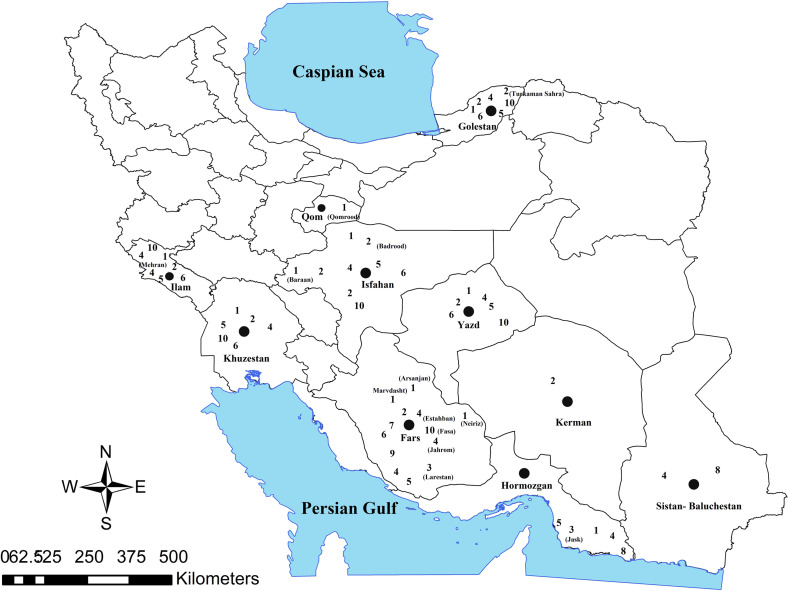
Distribution of cutaneous leishmaniasis reservoirs in Iran; **1**: *Meriones libycus*, **2**: *Rhombomys opimus*, **3**: *Gerbillus nanus*, **4**: *Tatera indica*, **5**: *Meriones persicus*, **6**: *Nesokia indica*, **7**: *Rattus norvegicus*, **8**: *Meriones hurrianae*, **9**: *Mus musculus*, **10**: *Rattus rattus*.

In Hormozgan province, in the south of Iran, *Gerbillus nanus* (Muridae) and *M. hurrianae* have been possible reservoirs of ZCL [15]. In Sistan and Baluchestan province, in the southeast of Iran, *M. hurrianae* and *T. indica* were found to be important hosts for *L. major* [62]. In Bahreman city, in Kerman province in the southeast of Iran, *R. opimus* was reported as a major reservoir for ZCL [40]. In Mehran city in Ilam province, in the west of Iran, *T. indica* was documented as a predominant reservoir for ZCL in rural areas [57, 95]; but in Khuzestan province, in the southwest of Iran, other rodents are responsible for *L. major* transmission [90]. In Isfahan province and rural areas of the Damghan city in Semnan province, in central Iran, *Nesokia indica* was recognized as a probable reservoir of ZCL [25, 108]. In Iran, *R. opimus*, *M. lybicus*, and *T. indica* are generally well-documented species for the *Leishmania* parasite [14, 88, 119, 138] (Table 1, Fig. 2).

**Table 1:** Reservoirs of cutaneous leishmaniasis in Iran.

References	Years	Focus	Reservoirs species	Etiological agent
[149]	1996	Badrood (Isfahan province)	*Meriones libycus, Rhombomys opimus*	*Leishmania major*
[116]	2001	Arsanjan (Fars province)	*Meriones libycus*	*Leishmania major*
[88]	2003	Marvdash (Fars province)	*Meriones libycus*	*Leishmania major*
[117]	2006	Neiriz city (Fars province)	*Meriones libycus*	*Leishmania major*
[84]	2007	Larestan (Fars province)	*T. indica*, *Gerbillus nanus*	*Leishmania major*
[115]	2007	Marvdasht (Fars province)	*Meriones libycus*	*Leishmania major*
[106]	2010	Golestan province	*Rhombomys opimus, Meriones libycus, Meriones persicus*	*Leishmania major*
[96]	2010	Fars province	*Rattus norvegicus*	*Leishmania major*
[83]	2011	Estahban (Fars province)	*Tatera indica*	*Leishmania major*
[86]	2011	Turkmen Sahra (Golestan province)	*Rhombomys opimus*	*Leishmania major*
[62]	2011	Sistan-Baluchestan Province	*Meriones Hurrianae, Tatera indica*	*Leishmania major*
[152]	2011	Qomrood (Qom province)	*Meriones libycus*	*Leishmania major*
[104]	2011	Fars province	*Mus musculus*	*Leishmania major*
[14]	2013	Jask (Hormozgan province)	*Tatera indica*, *Gerbillus nanus*, *Meriones persicus*, *Meriones hurrianae, Meriones libycus*	*Leishmania major*
[7]	2013	Golestan, Isfahan, Yazd, Fars, Khuzestan, and Ilam provinces	*Rhombomys opimus*, *Meriones libycus, Meriones persicus*, *Tatera indica, Nesokia indica, Rattus rattus*, *Mus musculus*	*Leishmania major*
[85]	2013	Fars province	*Meriones lybicus, M. persicus*	*Leishmania major*
[128]	2013	Baraan (Isfahan province)	*Rhombomys opimus, Meriones libycus*	*Leishmania major*
[30]	2014	Jahrom (Fars province)	*Tatera indica, M. Persicus*	*Leishmania major*
[40]	2014	Kerman province	*R. opimus*	*Leishmania major*
[90]	2017	Khuzestan province	*Tatera indica*	*Leishmania major*
[108]	2017	Fars province	*Nesokia indica*	*Leishmania major*
[95]	2018	Mehran (Ilam province)	*Tatera indica*	*Leishmania major*
[138]	2019	Isfahan province	*Meriones persicus, Nesokia indica*	*Leishmania major*

### Cutaneous leishmaniasis vectors

Like studies about reservoirs, there are many published articles on *Leishmania* vectors in Iran. The fauna of Iranian sandflies was first reported in 1930 [154]. *Phlebotomus* is known as the principal vector of CL in Iran. *Phlebotomus* includes 6 subgenera; i.e., subgenus *Adlerius*, *Euphlebotomus*, *Larroussius*, *Paraphlebotomus*, *Phlebotomus*, and *Synphlebotomus* [60]. Natural infections with *L. major* were shown in *P.* (*Phl.*) *papatasi* in all ZCL spots of Iran and was reported as the principal vector [13].

**Fig. 3:**
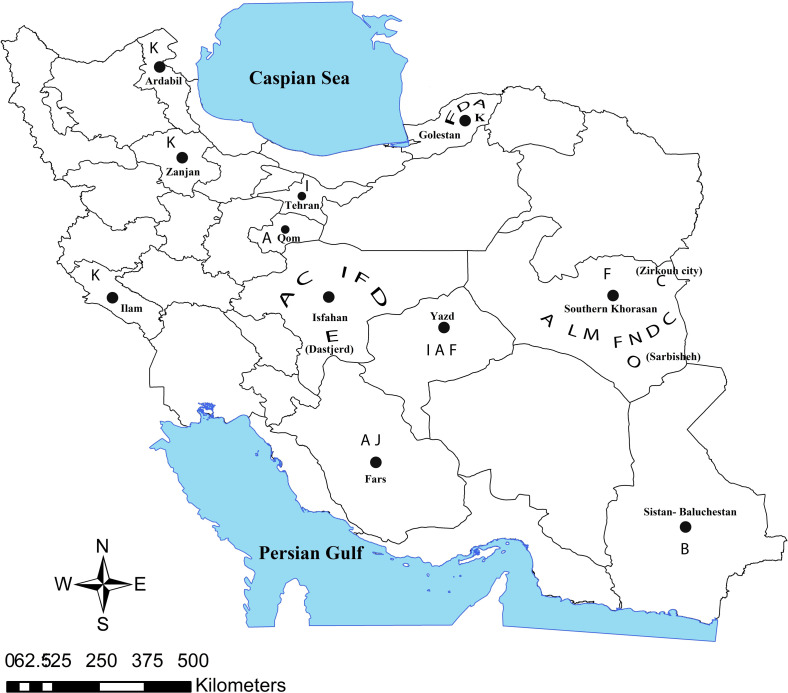
Distribution of anthroponotic cutaneous leishmaniasis in vectors in Iran; **A**: *Phlebotomus papatasi*, **B**: *Phlebotomus Salehi*, **C**: *Phlebotomus sergenti*, **D**: *Phlebotomus caucasicus*, **E**: *Phlebotomus mongolensis*, **F**: *Phlebotomus anderjevi*, **I**: *Phlebotomus ansarii*, **J**: *Phlebotomus alexandri*, **K**: *Phlebotomus perfiliewi*, **L**: *Phlebotomus jacusieli*, **M**: *Sergentomyia dentata*, **N**: *Sergentomyia sintoni*, **O**: *Sergentomyia clydei*.

**Fig. 4:**
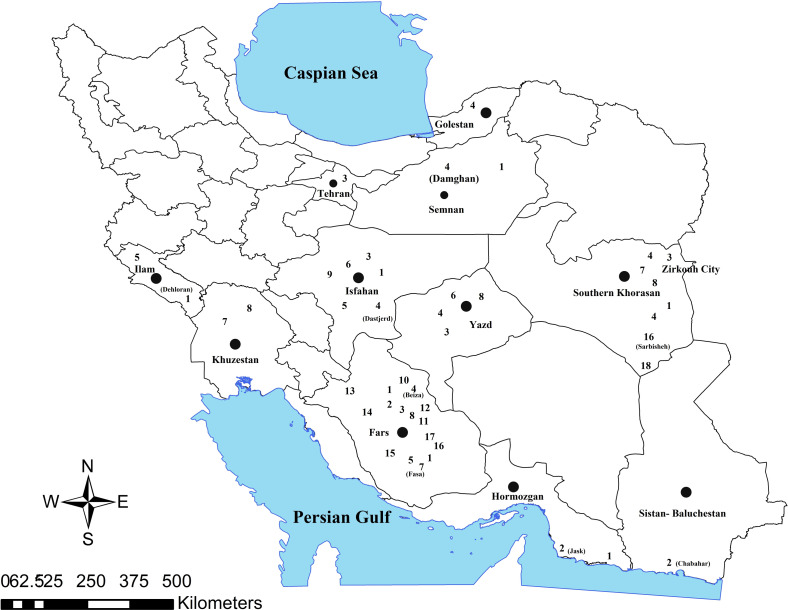
Distribution of zoonotic cutaneous leishmaniasis in vectors in Iran; **1**: *Phlebotomus papatasi*, **2**: *Phlebotomus Salehi*, **3**: *Phlebotomus sergenti*, **4**: *Phlebotomus caucasicus*, **5**: *Phlebotomus mongolensis*, **6**: *Phlebotomus ansarii*, **7**: *Phlebotomus alexandri*, **8**: *Sergentomyia dentata*, **9**: *Phlebotomus anderjevi*, **10**: *Phlebotomus tobbi*, **11**: *Sergentomyia theodori*, **12**: *Sergentomyia clydei*, **13**: *Sergentomyia Baghdadis*, **14**: *Sergentomyia squamipleuris*, **15**: *Phlebotomus bergeroti*, **16**: *Sergentomyia sintoni*, **17**: *Sergentomyia tiberiadis*, **18**: *Phlebotomus jacusieli*.

Other *Phlebotomus* species are considered probable vectors: *P.* (*Phl.*) *salehi* has been reported in Jask, in Sistan and Baluchestan province in the south of Iran, *P.* (*Syn.*) *ansarii* in Isfahan, and Yazd provinces, *P. caucasicus* in Isfahan, Damghan, Sarbisheh, Zirkouh, Yazd, and Fasa cities*, P. mongoliensis* in Isfahan, Ilam, and Fasa cities, *P. andrejevi* in Isfahan, *P. alexandri* in Khuzestan province, Zirkouh, and Fasa cities, and *P.* (*Phl.*) *bergeroti* in Fars province. *P.* (*Par.*) *sergenti* was demonstrated to be responsible for ACL in Khorasan-e Razavi, Isfahan, Yazd, Kerman, Fars, and Tehran provinces and in Zirkouh as well [16, 18, 54, 67, 68, 105, 114, 142, 148, 150] (Table 2, Figs. 3 and 4).

**Table 2:** Vectors of anthroponotic cutaneous leishmaniasis (ACL) and zoonotic cutaneous leishmaniasis (ZCL) Iran.

References	Years	Focus	Vectors species	Cl	
				ZCL	ACL
[154]	2012	Semnan province	*Phlebotomus caucasicus, Phlebotomus papatasi*	*	–
[60]	2013	Hormozgan province	*Phlebotomus papatasi, Phlebotomus salehi*	*	–
[150]	2015	Different endemic parts of CL in Iran	*Phlebotomus papatasi, Phlebotomus salehi, Phlebotomus sergenti, Phlebotomus caucasicus, Phlebotomus mongoliensis, Phlebotomus andrejevi, Phlebotomus ansarii, Phlebotomus alexandri, Phlebotomus Perfiliewi*	*	*
[13]	2013	Beiza District (Fars province)	*Phlebotomus papatasi, Phlebotomus. sergenti, Phlebotomus tobbi, Phlebotomus salehi, Phlebotomus caucasicus, Sergentomyia theodori, Sergentomyia clydei, Sergentomyia dentate, Sergentomyia baghdadis, Sergentomyia squamipleuris*	*	–
[105]	2013	Fars province	*Phlebotomus papatasi, Phlebotomus bergeroti, Phlebotomus mongoliensis, Phlebotomus tobbi, Sergentomyia sintoni, Sergentomyia tiberiadis*	*	–
[113]	2013	Sarbisheh (Southern Khorasan province)	*Phlebotomus papatasi*, *Phlebotomus sergenti*, *Phlebotomus caucasicus*, *Phlebotomus mongoliensis*, *Phlebotomus jacusieli, Sergentomyia dentata*, *Sergentomyia sintoni, Sergentomyia clydei.*	*	*
[54]	2013	Yazd province	*P. sergenti, Phlebotomus sergenti; Phlebotomus caucasicus, Phlebotomus caucasicus, Phlebotomus Ansari, Phlebotomus ansari, Sergentomyia dentate, Sergentomyia dentate*	*	–
[16]	2016	Fasa (Fars province)	*Phlebotomus papatasi*	*	–
[67]	2017	Dehloran (Ilam province)	*Phlebotomus papatasi*	*	–
[18]	2017	Zirkouh City (Southern Khorasan province)	*Phlebotomus sergenti*	–	*

### Effects of meteorological and ecological characteristics on CL

Control and surveillance of leishmaniasis are not possible unless all environmental factors are measured together with the identification of reservoirs and vectors. Therefore, systems such as GIS have been designed to predict incidence based on spatial and temporal components of CL. Climatic factors (temperature, precipitation, and relative humidity) can establish a different pattern of CL occurrence in two nearby cities such as Gilan-e-Gharb and Kermanshah in the west of Iran [43]. Temperature had a significant relationship with the incidence rate of CL in Isfahan province: sandflies were more active in dry or moderate seasons [112]. Thus, most cases in Iran are detected from September to December [8, 74]. During these months, the temperature reaches 23–27 °C, which is suitable for the reproduction of sandflies. On the other hand, emergence of sandfly species in hot months affects workers who have seasonal occupations [20].

On the contrary, in the west of Iran, some peaks of CL incidence have been shown in winter, reported in January and February after an incubation period of the disease [63, 64]. The burden of CL has different dynamics in Iran. In the northeast of Iran, rainfalls and floods demolish the breeding sites of sandflies; leading to a drop in CL [139]. In contrast, in the southwest of Iran, wet days and humidity significantly increase the incidence of CL cases [12]. CL cases were mostly found in low-altitude areas in the south, center, northeast, and west of Iran [93]. For example, in Mehran and Dehloran (Ilam province), CL is strictly spread at an elevation of fewer than 215 m [153].

Some disasters such as an earthquake can change sleeping patterns and increase CL incidence because people have to stay outside in an open space. This model was presented in Dehloran city, in the west of Iran, after the occurrence of an earthquake in 2015 [98]. Jagin Dam provided suitable ecological conditions for *T. indica* and *P. papatasi* due to agricultural activities [37]. Notably, *Rhombomys opimus* (great gerbil) as a diurnal rodent spends 2/3 of its time outside of the burrow for feeding and this behavior can cause intensive close contact with vectors. Another factor is vegetation, which leads CL to be more prevalent in landscapes with low vegetation cover in the northeast of Iran [92].

### *Leishmania* parasite identification methods

*Leishmania* species are responsible for the spectrum of clinical manifestations or different epidemiologic features; thereby, specific detection of parasites is necessary to understand their complexity. Staining smears by Giemsa under microscope and cultivation were the common diagnostic tests for early detection for a long time. The sensitivity of parasitological diagnostic tests is less than 70%, which makes it necessary to have access to a large number of parasites in biopsy and experienced personnel. In contrast, the culture of promastigotes is a prolonged protocol and contamination is a concerning issue, but both are weak in the identification of *Leishmania* species [47].

Serological tests are not used extensively in CL diagnosis as their specificity and sensitivity fluctuate and have cross-reaction with trypanosomatid parasites [121]. The isoenzyme electrophoresis (IE) technique is considered the gold-standard method for the identification of *Leishmania* [101]. Molecular methods such as PCR are still used to detect the specific DNA of *Leishmania* and this is a well-founded source for identifying *Leishmania* species. DNA fragments such as the small subunit rRNA (SSU rRNA) gene, repetitive sequences, microsatellite DNA, splice leader mini-exon (SLME), the beta-tubulin gene region, the gp63 gene locus, mini-exon-derived RNA genes, internal transcribed spacer (ITS) regions of the rRNA genes, and kinetoplast DNA (kDNA), SLME, kDNA, and ITS1 are used in PCR assays for the diagnosis of *Leishmania* parasites isolated from microscopic and culture samples. As a result, the kDNA PCR is the best diagnostic method in terms of sensitivity for *L. major* and *L. tropica* [24, 41, 81, 124]. Different studies about *Leishmania* identification methods in human cases carried out in Iran are summarized in Table 3.

**Table 3:** Different *Leishmania* identification methods in human cases carried out in Iran.

References	Years	Focus	Recognized method	Parasite species	DNA
[7, 86]	2011, 2013	Isfahan, Golestan	Nested PCR	*L. major, L. gerbilli*	ITS1-5.8S rRNA-ITS2
[86, 119]	2011	Golestan	Nested PCR	*L*. *major*, *L*. *turanica*	ITS1-5.8S rRNA-ITS2
[32]	1981	Isfahan	IFAT^1^	*Leishmania sp.*	–
[11]	2000	Fars	ELISA^2^, IFAT	*L*. *major*, *L*. *tropica*	–
[51]	2005	Fars	Isoenzyme	*L*. *major*, *L*. *tropica*	–
[80]	2006	Kashan	PCR-SSCP^3^	*L. major*	ITS^7^ (ITS1, ITS2)
[39]	2007	Mirjaveh	Smears, Culture, PCR^4^	*Leishmania sp.*	kDNA^8^
[146]	2009	Mashhad	PCR-RFLP^5^	*L*. *major*, *L*. *tropica*	Mini-exon gene
[78]	2010	Mashhad	Culture, PCR	*L. major*, *L. tropica*	kDNA
[34]	2010	Shiraz	PCR	*L*. *major*, *L*. *tropica*	kDNA
[109]	2010	Shiraz	Smears, Culture, PCR	*L. major*, *L. tropica*	kDNA
[49]	2010	Kermanshah	Culture, PCR-RAPD^6^	*L. major*	ITS1
[31]	2011	Isfahan, Bam	PCR-RFLP	*L. major*, *L. tropica*	ITS1
[35]	2011	All endemic areas	Smears, PCR	*L. major*, *L. tropica*	kDNA
[131]	2011	Khuzestan province	PCR-RFLP	*L. major*, *L. tropica*	Mini-exon gene
[137]	2012	Kashan	Culture, PCR-RFLP	*L. major*, *L. tropica*	ITS1, KDNA
[17]	2012	Fars province	PCR	*L*. *major*, *L*. *tropica*	ITS1
[50]	2012	Isfahan	Culture	*L. major*, *L. tropica*	ITS1
[29]	2012	Isfahan, Ahwaz	PCR-PPIP, PCR-RFLP	*L. major*	ITS
[79]	2012	All endemic areas	PCR-RAPD	*L. major*, *L. tropica*	KDNA
[120]	2013	Qom province	PCR	*L*. *major*	ITS1
[46]	2013	Lorestan Province	Nested-PCR	*L. major*, *L. tropica*	kDNA
[73]	2103	Lorestan Province	Smears, Culture, PCR	*L. major*, *L. tropica*	ITS
[127]	2013	Khorasan province	Culture, PCR-RAPD	*L. tropica*	kDNA
[135]	2013	Fasa	PCR	*L. major*, *L. tropica*	kDNA
[33]	2014	Yazd	PCR-RFLP	*L*. *major*, *L*. *tropica*	ITS1
[144]	2014	Isfahan	PCR	*L. major*	ITS1
[134]	2015	Sarakhs	Smears, PCR	*L. major*, *L. tropica*	kDNA
[3]	2015	Khorasan province	Smears, PCR	*L. major*, *L. tropica*	kDNA
[53]	2016	Shiraz, Isfahan	Nested-PCR	*L*. *major*, *L*. *tropica*	kDNA
[140]	2016	Chabahar	PCR	*L. major*, *L. tropica*	kDNA
[69]	2017	Ilam province	PCR-RFLP	*L*. *major*	ITS1
[123]	2017	Gonabad, Bardaskan, Kashmar	PCR	*L*. *major*, *L*. *tropica*	kDNA
[23]	2017	Varamin	PCR-RFLP	*L. major*, *L. tropica*	ITS1
[42]	2018	Bam, Kerman, Shiraz	PCR-RFLP	*L*. *major*, *L*. *tropica*	kDNA
[19]	2018	Kerman and Bam	Culture, PCR	*L. tropica*	ITS1, 7SL RNA, Hsp70
[155]	2019	Sistan and Baluchestan	Culture, PCR-RFLP	*L. major*, *L. tropica*	ITS

1 Immunofluorescence antibody test (IFAT).

2 Enzyme-linked immunosorbent assay (ELISA), PCR-SSCP.

3 Polymerase chain reaction single-strand conformation polymorphism (PCR-SSCP).

4 Polymerase chain reaction (PCR).

5 Polymerase chain reaction-restriction fragment length polymorphism (PCR-RFLP).

6 Polymerase chain reaction random amplified polymorphic DNA (PCR-RAPD)

7 Internal transcribed spacer (ITS).

8 kinetoplast DNA (kDNA).

## Conclusion

Our findings showed that cutaneous leishmaniasis has various patterns in Iran. There is heterogeneity in leishmaniasis in different parts of the country based on molecular identifications. KDNA and ITS1 fragments have mostly been used in genomic diagnostic procedures, while KDNA is a favorite targeted region for amplifying by PCR. Travel has been a hallmark of the distribution of *Leishmania* in Hamedan and Ilam (in the west) and South Khorasan provinces of Iran. In Iran, *R. opimus*, *M. lybicus*, and *T. indica* were well-documented species for hosting the *Leishmania* parasites. In Khorasan Razavi province, Kerman, Yazd, and sometimes in Shiraz and Tehran cities, *L. major* is present, but *L. tropica* is the predominant parasite.

The prevalence of *R. opimus* extends from Turkmen Sahra in the northeast to Isfahan in center of Iran with co-infection of *L. major* and *L. turanica*. More studies are required to clarify the potential role of *L. turanica* in CL. Furthermore, *L. gerbilli* has been identified in some vectors, such as *P. papatasi*, *P. sergenti*, *P. caucasicus*, *P. mongoliensis*, *P. andrejevi*, and *P. ansarii*. The relationship schema between vectors and reservoirs is complicated. Some sandflies play the role of common vectors concerning different parasite species. Although cutaneous leishmaniasis has been a predicament for the health system, it is generally controlled in Iran. Spraying, insecticide-treated nets (ITNs), and improvement in social services in the suburban community can reduce the problems associated with CL.
